# Evaluation of myocardial dispersion of repolarization in patients with heart transplantation

**DOI:** 10.3906/sag-1807-90

**Published:** 2019-02-11

**Authors:** Cengiz BURAK, Erkan BAYSAL, Muhammed SÜLEYMANOĞLU, Çağrı YAYLA, Serkan CAY, Ümit KERVAN

**Affiliations:** 1 Department of Cardiology, Mardin State Hospital, Mardin Turkey; 2 Department of Cardiology, Diyarbakır Gazi Yaşargil Training and Research Hospital, Diyarbakır Turkey; 3 Department of Cardiology, Bingöl State Hospital, Bingöl Turkey; 4 Department of Cardiology and Cardiovascular Surgery, Türkiye Yüksek İhtisas Training and Research Hospital, Ankara Turkey

**Keywords:** Tp-e/QTc ratio, heart transplantation, myocardial dispersion of repolarization

## Abstract

**Background/aim:**

The number of patients with heart transplantation has dramatically increased in the last decade. Considerable studies have suggested that the interval from the peak to the end of the electrocardiographic T wave (Tp-e) may correspond to the transmural dispersion of repolarization and increased Tp-e interval and Tp-e/QT ratio are associated with malignant ventricular arrhythmias. We analyzed the dispersion of myocardial repolarization using electrocardiographic Tp-e interval and Tp-e/QTc ratio in patients with heart transplantation.

**Materials and methods:**

This observational study included 38 patients (12 female and 26 male) with heart transplantation and 38 well-matched controls.  From electrocardiograms, Tp-e interval and Tp-e/QTc ratio were calculated and compared between the 2 groups.

**Results:**

Noninvasive arrhythmia indicators including Tp-e interval (84.63 ± 14.17 ms vs 71.82 ± 7.47 ms, P < 0.001), Tp-e/QTc ratio (0.19 ± 0.04 vs 0.16 ± 0.02, P < 0.001) and QTc interval except QT interval were significantly higher in transplanted hearts compared to normal hearts.

**Conclusion:**

Patients with heart transplantation have increased myocardial dispersion of repolarization.

## 1. Introduction

Despite advances in pharmacologic and device treatment of chronic heart failure, long-term morbidity and mortality remain high and many patients progress to end-stage heart failure. Over the last 5 decades, heart transplantation (HTx) has become the preferred therapy for selected patients with end-stage heart disease. However, arrhythmias are increasingly recognized and significantly affecting quality of life, morbidity, and survival. Many parameters that predict the risk for ventricular arrhythmia (VA) in these patients can be assessed using surface ECG. Tp-e interval indicating indirect measurement of transmural dispersion of repolarization is an easily measurable parameter, which has been attributed to arrhythmogenesis (1,2). To the best of our knowledge, there is no data regarding such arrhythmia indicators in patients with heart transplantation. Therefore, we aimed to investigate noninvasive ECG arrhythmia parameters in transplanted hearts.

## 2. Materials and methods

### 2.1. Study population

This is a cross-sectional study that included patients with heart transplantation between 2008 and 2012 in Türkiye Yüksek İhtisas Training and Research Hospital. We evaluated 38 patients having a median transplantation time of 4 years. Well-matched 38 healthy subjects were also included. Medical network database was reviewed to obtain medical records regarding demographic, clinical and laboratory characteristics of the study population. Heart transplant patients underwent the bicaval anastomosis technique for orthotopic heart transplantation with a median total ischemia time <4 h. No rejection sign was observed in biopsies which were periodically taken at the first, third, sixth, and twelfth months. Through the first year after transplantation; cyclosporine, mycophenolat mofetil, and steroid were given to the patients. In the case of renal deterioration, everolimus was chosen instead of cyclosporine. Exclusion criteria were as follows: age <18 years, marked inflammation, hypo- or hyperthyroidism, severe pulmonary disease, coronary artery disease, hepatic/renal failure, use of drugs prolonging QT interval, antiarrhythmic drugs other than beta blocker, atrial fibrillation/flutter or paced rhythm, complete bundle brunch block, rejection findings, and severe left ventricular dysfunction shown in echocardiography and myocardial biopsy. All patients provided written informed consent. The Institutional Review Board approved the study.

### 2.2. ECG parameters

The standard 12-lead electrocardiograms with the paper speed of 25 mm/s, amplitude of 10 mm/mV, and filter range of 0.15–100 Hz were used and, to increase the sensitivity of the measurements, the obtained ECGs were transferred to computer and analyzed with a magnification of 400%. The QT interval was measured from the beginning of the QRS complex to the end of the T wave where the T wave returns to the isoelectric line when available. In unavailable cases, the end of the T wave was determined as the intercept between the isoelectric line and the tangential line drawn through the maximum slope of the T wave (3). The Tp-e interval was defined as the interval from the peak of T wave to the end of the T wave where it returns to the isoelectric line when available. In unavailable cases, the previously described method was used(3).

For all transplanted patients, posttransplantation first year ECGs were analyzed. In this way, the QT and Tp-e intervals were measured in leads V2, V6, and DII using subsequent 3 beats by two independent cardiologists. When there was a difference of more than 20 ms between the measurements, a third cardiologist analyzed the tracing. Subsequently, the mean value of data was determined for the aforementioned leads. The QT interval was corrected using the Bazzett’s formula (4). The Tp-e/QTc ratio was calculated using the previously measured parameters.

### 2.3. Statistical analysis 

Data were analyzed with the SPSS software version 20.0 for Windows (SPSS Inc., Chicago, IL, USA). Categorical variables were presented as frequency and percentage. The χ2 test and Fisher’s exact test were used to compare the categorical variables. The Kolmogorov–Smirnov test was used to assess the distribution of the continuous variables. Student’s t-test was used for variables with normal distrib­ution and the values were presented as mean ± SD. Continuous variables without normal distribution were analyzed using the Mann–Whitney U test and the obtained values were presented as median (50th) values and interquartile ranges (25th and 75th). A two-tailed P-value of <0.05 was considered statistically significant. Interobserver and intraobserver variability were found to be less than 5%.

## 3. Results

We enrolled 38 consecutive transplanted patients [26 males (68.4%); mean age, 40.2 ± 15.1 years] and 38 healthy control subjects [24 males (63.2%); mean age, 42.2 ± 13.6 years]. The baseline characteristics and echocardiographic and laboratory outcomes of the study groups were presented in Table 1. Baseline characteristics including age, sex, and BMI were similar between the 2 groups. The mean LVEF was significantly higher in control group (59.0 ± 6.1 vs 62.6 ± 2.1, P = 0.010). Furthermore, there were statistically significant differences regarding the hemoglobin levels between the two groups (12.1 ± 2.0 mg/dL vs 14.6 ± 1.2 mg/dL, P < 0.001)

**Table 1:** Baseline characteristics and echocardiographic and laboratory parameters.

	Transplantedpatients (N = 38)	Controlsubjects (N = 38)	P-value
Male, n (%)	26 (68.4)	24 (63.2)	0.629
Smoking, n (%)	2 (5.39)	4 (10.5)	0.674
Diabetes mellitus, n (%)	7 (18.4)	2 (5.3)	0.153
Hypertension, n (%)	6 (84.2)	7 (18.4)	0.761
Hyperlipidemia, n (%)	8 (21.1)	3 (7.9)	0.191
Age, years	40.2 ± 15.1	42.2 ± 13.6	0.543
BMI (kg/m2)	24.6 ± 4.7	26.2 ± 2.6	0.078
LVEF (%)	59.0 ± 6.1	62.6 ± 2.1	0.010
Hemoglobin, (g/dL)	12.1 ± 2.0	14.6 ± 1.2	<0.001
Potassium (mmol/L)	4.4 ± 0.4	4.2 ± 0.3	0.072
Creatinine (mg/dL)	1.16 ± 0.3	0.93 ± 0.23	0.002
Glucose (mg/dL)	103 ± 22	98.2 ± 20	0.063
HDL-cholesterol, (mg/dL)	45.1 ± 13.3	53.1 ± 13.7	0.931
LDL-cholesterol, (mg/dL)	90.1 ± 44.2	119.1 ± 32.9	0.344
Triglyceride, (mg/dL)	155.1 ± 77.4	136.7 ± 73.9	0.740

Data are given as mean ± SD or %. BMI, body mass index; LVEF, left ventricular ejection fraction; HDL, high-density lipoprotein; LDL, low-density lipoprotein

The ECG measurements were given in Table 2. The mean heart rate of patients with heart transplantation was 94.53 ± 13.27 bpm, which was higher compared to the control group (81.08 ± 11.34 bpm). The mean QTc, Tp-e, and Tp-e/QTc values were significantly higher in the study group compared to the control group (Figure). As compared with the control group, Tp-e/QTc ratio was significantly higher in leads V6 and DII (P < 0.005) and there was a trend to be higher in lead V2 (P = 0.06) 

**Table 2:** Electrocardiographic parameters of the study population.

	Transplanted patients	Control subjects	P value
Heart rate (bpm)	94.53 ± 13.27	81.08 ± 11.34	<0.001
QT interval in lead DII (ms)	351.00 ± 52.12	346.21 ± 30.46	0.627
QT interval in lead V2 (ms)	354.32 ± 50.03	345.82 ± 29.42	0.370
QT interval in lead V6 (ms)	356.21 ± 54.14	344.66 ± 28.65	0.412
Tp-e interval in lead DII (ms)	82.53 ± 8.78	64.47 ± 8.78	<0.001
Tp-e interval in lead V2 (ms)	84.63 ± 14.17	71.82 ± 7.47	<0.001
Tp-e interval in lead V6 (ms)	89.29 ± 13.98	71.34 ± 6,65	<0.001
QTc interval in lead DII (ms)	438.2 ± 67.4	399.9 ± 27.4	0.002
QTc interval in lead V2 (ms)	442.3 ± 64.6	399.5 ± 27.4	<0.001
QTc interval in lead V6 (ms)	444.7 ± 66.2	398.4 ± 28.1	<0.001
Tp-e/QTc ratio in lead DII	0.19 ± 0.04	0.16 ± 0.02	<0.001
Tp-e/QTc ratio in lead V2	0.20 ± 0.04	0.18 ± 0.02	0.062
Tp-e/QTc ratio in lead V6	0.20 ± 0.04	0.18 ± 0.02	0.001

Data are given as mean ± SD.

**Figure 1:**
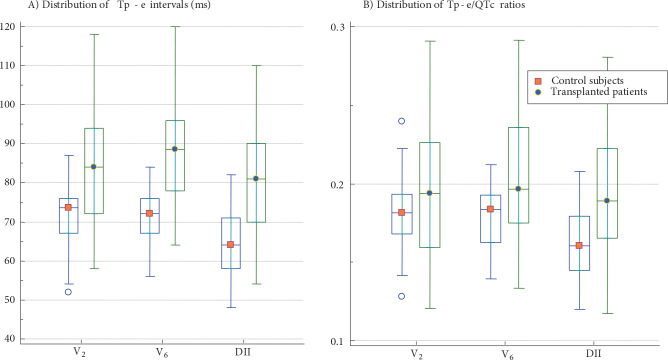
Box plots for Tp-e intervals and Tp-e/QTc ratios. Panel A shows Tp-e intervals, Panel B shows Tp-e/QTc ratios in the heart transplant patients and the control subjects. The horizontal line inside each box indicates the median, the top and bottom of the box indicate the interquartile range, the I bars indicate the 5th and 95th percentiles, and the circles indicate outliers. Noninvasive arrhythmia indicators including Tp-e interval and Tp-e/QTc ratio are significantly higher in the transplanted hearts compared to the normal hearts.

## 4. Discussion

In the literature, there are no studies that have examined the predictors of VA in transplanted patients. In the current study, we detected that electrocardiographic repolarization parameters were significantly increased in the heart-transplant patients compared to the control subjects.

It has been shown that there are three different types of electrophysiological cells in the ventricular myocardium (endocardial, epicardial, and midmyocardial cells), which are effective during the distinct phases of repolarization throughout the generation of T wave in ECG (5). If an isolated ventricular sample is analyzed, it is possible to see that the time from the peak to the end of the T wave respectively corresponds to the repolarizations of the epicardial and midmyocardial cells. Therefore, Tp-e interval is a measurement that reflects the transmural dispersion of repolarization (6). In swine heart, Xia et al. showed that the measurement of Tp-e interval in precordial leads might indicate the total (transmural, apicobasal, and global) dispersion of repolarization (7). However, the other studies have pointed out that Tp-e interval does not completely show the transmural dispersion of repolarization (8). Instead, the transmural dispersion of repolarization index can be used to predict malignant arrhythmias (5,9). This proposal has also been supported by previous studies related to hypertrophic cardiomyopathy, Brugada syndrome, congenital or acquired long QT syndrome, and other disorders concerning pathophysiological conditions related to the heterogeneity of repolarization (5,10,11). In heart transplant patients, the significance of Tp-e interval and Tp-e/QTc ratio have not been studied prospectively and the real frequency of arrhythmic episodes is unclear. 

Unlike the Tp-e interval, the Tp-e/QTc ratio is an independent parameter and it is more important in predicting the risk. Letsas et al. revealed that together with the Tp-e interval, the Tp-e/QTc ratio is a strong predictor of ventricular tachycardia/ventricular fibrillation in Brugada syndrome (12). In our study, we particularly paid attention to the Tp-e/QTc ratio and showed that there was a statistically significant difference.

There are several proposed mechanisms for predisposition to the development of arrhythmia in heart transplant patients. In this context, the longer ischemia time for the graft can cause damage in the conduction system during the early or late postoperative period. Perioperative ischemic damage resulted in endocardial fibrosis which has likely major mechanical role in the majority of cases(13). Although the most commonly used method is the bicaval transplantation technique, the bi-atrial anastomosis technique is also preferred in some centers even if it may give rise to undesired results such as atrial rhythm and conduction disturbances. 

Donor heart has complete denervation. With the absence of parasympathetic activity, the mean resting heart rate is higher and the heart rate variability reduces in transplant patients compared to controls. Due to being dependent on the body weight and heart rate, QT and Tp-e intervals are less predictive in determining the arrhythmogenesis. In this perspective, when compared to one of these intervals, the Tp-e/QTc ratio is preferred. This is mainly due to the fact that the Tp-e/QTc ratio remains steady despite the dynamical variations in heart rate (14). In our study, the mean heart rate was found to be higher in heart transplant patients. By considering all these results, we concluded that the Tp-e/QTc ratio is a crucial parameter in the evaluation of transmural dispersion of repolarization.

As is known, the cardiac allograft vasculopathy is relatively more frequent and it is one of the pivotal prognostic indicators in the late period of heart transplantation. The ischemia resulting from the vasculopathy or atherosclerosis can be a trigger of ventricular arrhythmia (15). Nonspecific late-stage graft failure and rejection are other conditions that generate predisposition to arrhythmia (16). Similarly, it was shown that arrhythmias were related to worse clinical outcomes (17). In general, it is thought that ventricular arrhythmia in the heart transplant patients may be related to graft rejection or failure (18). In consistence with the literature, we have shown that these patients have increased myocardial dispersion of repolarization which can result in arrhythmic death and graft failure.

Early diagnosis and management of these problems could facilitate life standards adequately. Therefore, we think that it is essential to identify the individual risk stratification according to the therapeutic strategy and complications in order to prevent arrhythmia.

There are some limitations to our study. Since heart transplantations are rare and special cases, the number of patients analyzed here was relatively small. In addition, it may be inconvenient to generalize our results because it is not a multicenter, randomized, and very comprehensive, but a retrospective study. Finally, the inability of the short- and long-term mortality and morbidity follow-ups and the lack of arrhythmic outcome were important restrictions.

To conclude, in the current study, we showed that Tp-e interval and Tp-e/QTc ratio in patients with heart transplantation were significantly higher. Even though the present study did not cover all the relevant questions, it is crucial in drawing attention particularly to predictors of arrhythmia in heart transplant patients.
